# Concomitant Fracture Radial Head and Shaft With Dislocation of the Distal Radioulnar Joint: A Rare Variant of the Essex-Lopresti Lesion

**DOI:** 10.5435/JAAOSGlobal-D-22-00042

**Published:** 2022-08-08

**Authors:** Rajiv Kaul, Chetan Sood, Neha Akhoon

**Affiliations:** From the Military Hospital Dehradun, Dehradun, India (Dr. Kaul and Dr. Akhoon), and the Department of Orthopaedics, Armed Forces Medical College, Pune, India (Dr. Sood).

## Abstract

The Essex-Lopresti lesion is a challenging injury both for diagnosis and management. These often tend to be missed, resulting in incapacitating pain and joint stiffness despite treatment. In rare circumstances, they may occur in association with other injuries of the forearm or elbow. We describe an atypical Essex-Lopresti variant comprising a radial head and shaft fracture with an associated distal radioulnar joint dislocation in a 36-year-old man.

The classic Essex-Lopresti lesion, which comprises a fracture of the radial head, disruption of the interosseous membrane, and a dislocation of the distal radioulnar joint (DRUJ), was first introduced by Curr and Coe^1^ in 1946 and later described and named after Peter Essex-Lopresti^2^ in 1951. Another eponymous fracture, named after Italian surgeon, Riccardo Galeazzi,^3,4^ comprises a fracture of the radial shaft along with disruption of the DRUJ. The mechanism of injury in both these injuries is similar, primarily, an axially directed force along the long axis of the forearm, which results in a traumatic dislocation of the DRUJ, disruption of the interosseous membrane, and with continued force, a compression type fracture or dislocation of the radial head. A few rare variants of the abovementioned patterns have been described in the literature, although the combination of a Galeazzi fracture and an Essex-Lopresti lesion has been reported only twice. We report a distinct and rare variant of the abovementioned combination which comprised a concomitant radial head and shaft fracture along with a DRUJ dislocation.

## Case Report

**Figure 1:**
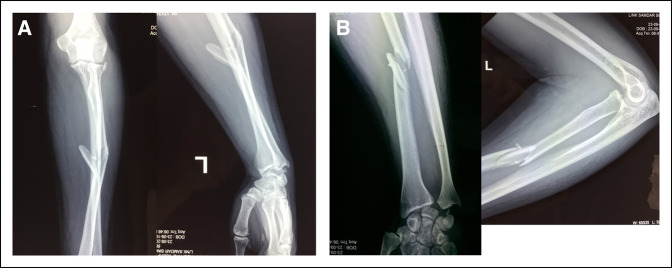
**A**, Preoperative radiographs showing the anteroposterior view. **B**, Preoperative radiographs showing the lateral view.

**Figure 2:**
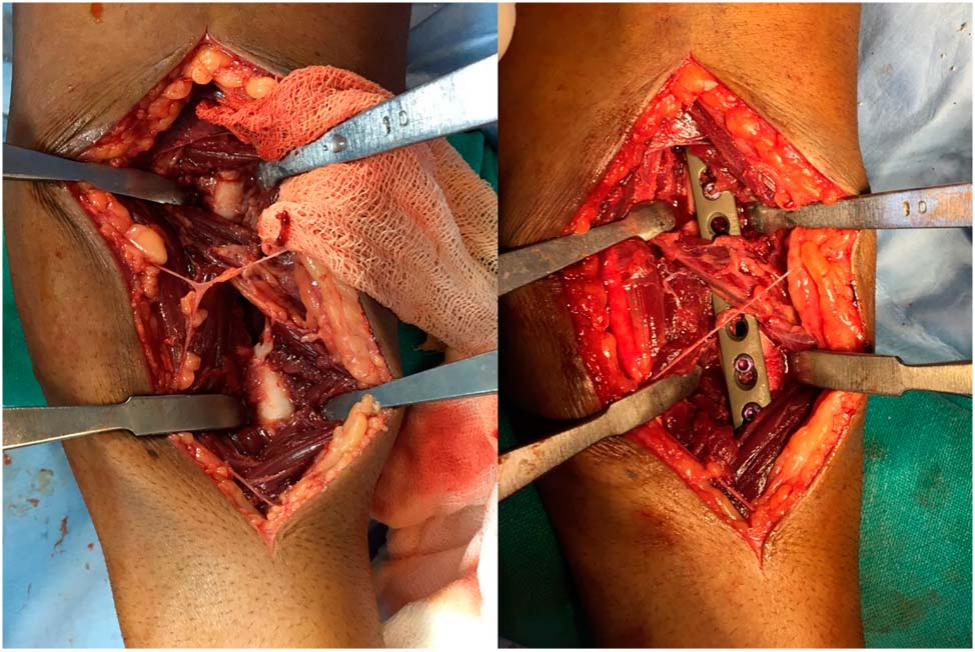
Intraoperative images before and after fixation of the radial shaft.

**Figure 3:**
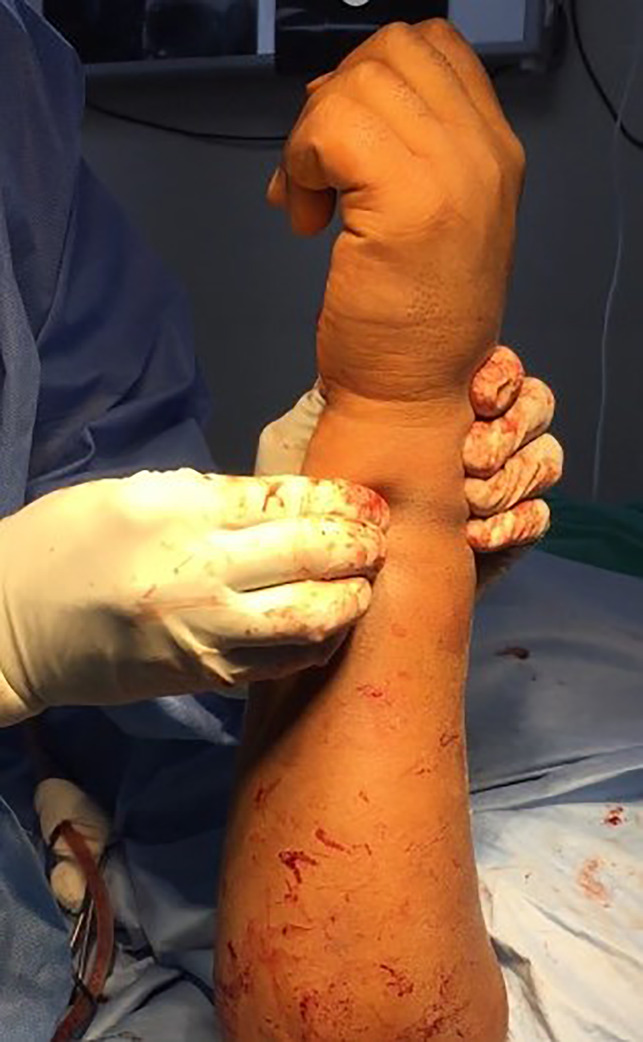
Image demonstrating the ballottement test for stability of the distal radioulnar joint.

A 36-year-old male soldier sustained an injury to his left forearm because of a motor vehicle accident and presented with a swollen and painful forearm with restricted movements of the wrist and elbow. Radiographs showed a comminuted fracture of the radial shaft and head along with a DRUJ dislocation (Figure 1, A and B). After ruling out compartment syndrome and subsidence of the edema, the patient was taken up for surgery. Under regional anesthesia, a standard volar approach to the radial shaft was used. Intraoperatively, the interosseous membrane was found to be disrupted from its radial attachment sparing the multifragmentary segment. After restoring the length and axial alignment of the shaft, a bridge plating was done (Figure 2) with careful preservation of the soft-tissue attachments of the intermediate segment. Next, a stability check was done for the DRUJ, which was unstable and ballotable (Figure 3).

**Figure 4:**
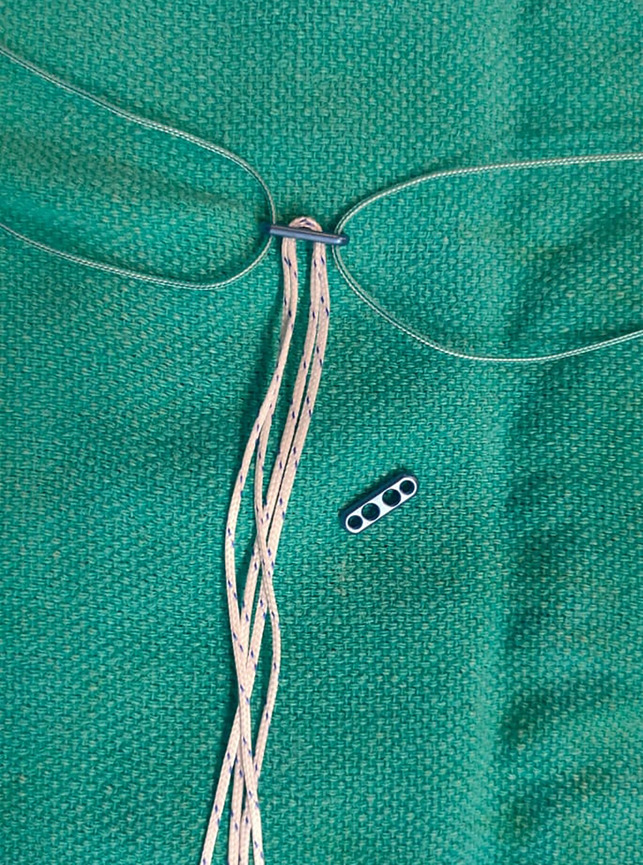
Image showing double endobutton suspensory construct using a double suture loop.

**Figure 5:**
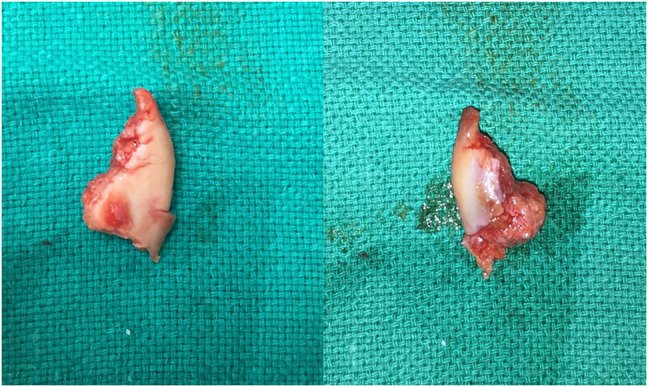
Images showing a partially excised radial head.

**Figure 6:**
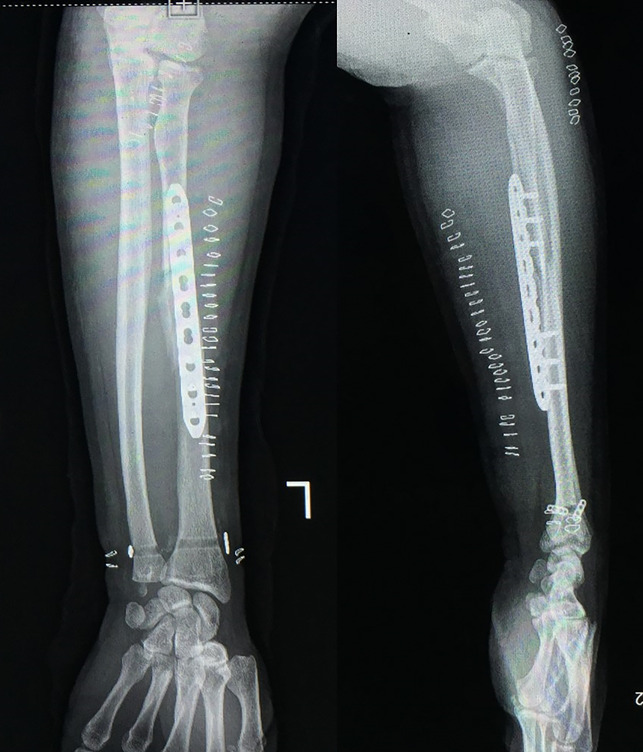
Postoperative radiographs showing anteroposterior and lateral views.

A suspensory fixation of the DRUJ using a double endobutton construct was conducted in the following manner. First, a stab incision was made around 1.5 cm proximal to the ulnar styloid over the lateral aspect of the distal ulna. After reducing the DRUJ, a 1.6-mm Kirschner wire was passed from the ulna to the radius, perpendicular to the shafts of both bones, proximal to the DRUJ. Once accurate reduction was confirmed in anteroposterior and lateral views, a bony tunnel was created using a 2.5-mm cannulated drill bit. A suspensory construct was prepared using two 2-mm endobuttons, with a high–tensile strength suture loop (Figure 4). A Beath pin was used to retrieve the suture ends from the radial side. A small 0.5-cm incision was made over the exit wound of the Beath pin, and soft tissues were bluntly dissected down to the bone to create space for seating the button. The loop construct was advanced and flipped on the ulnar side under direct vision so as to avoid tendon entrapment beneath the button. The radial-sided button was introduced; the suture ends were threaded through its central holes; and a sliding knot was placed after adequate tensioning of the loop to create an elastic, suspensory fixation. The ballottement test confirmed a stable fixation of the joint. Finally, a standard Kocher approach was used to conduct a partial excision of the radial head, which was a tiny fragment along the nonarticulating zone of the head (Figures 5 and 6). The capsule and lateral collateral ligament were repaired at the end of the procedure. The elbow was splinted in supination for three weeks to protect the repair.

Prophylactic indomethacin was given to minimize chances of Heterotopic ossification. Active range of motion (ROM) was commenced at 4 weeks. The patient was reviewed at 3-month intervals for a total of 18 months to look for progress of union and to reassess the stability of the joints.

## Results

**Figure 7:**
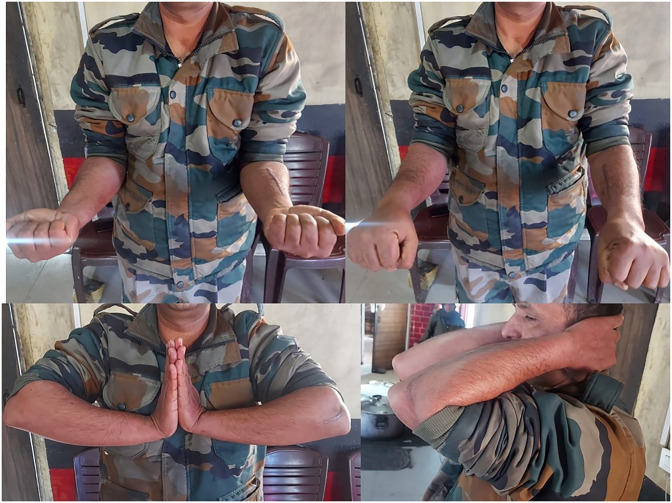
Images demonstrating functional range of motion at 18 months.

**Figure 8:**
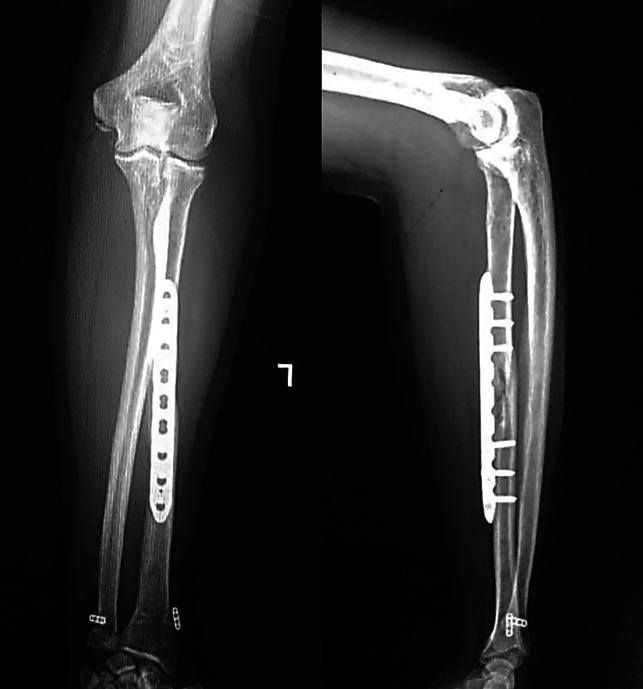
Eighteen-month follow-up radiographs showing anteroposterior and lateral views.

The quick Disability of Shoulder, Arm, and Hand score improved from 87 preoperatively to 11 at the final follow-up. His ROM was 0° to 150° of elbow flexion, 0° to 90° of supination, and 0° to 80° of pronation (Figure 7). Radiologically, there was complete union of the fracture with a normal relationship of proximal and DRUJs (Figure 8). The bony tunnel for the fixation had completely healed without any evidence of synostosis. Clinically, there was no collateral laxity or DRUJ instability. The individual was able to perform his bonafide duties including handling of arms and ammunition.

## Discussion

The Essex-Lopresti injury is a rare and commonly missed entity.^5^ It results from a longitudinal force transmitted along the axis of the forearm, disrupting the interosseous membrane and the DRUJ resulting in a fracture of the radial head. It is not uncommon to come across a delayed presentation, which usually manifests as ulnar-sided wrist pain due to ulnocarpal abutment consequent to a positive ulnar variance. These injuries were further classified into three subtypes based on the amount of comminution and station of the radial head by Edwards and Jupiter^6^ in 1988. Hargadon and Porter,^7^ in 1988, described a rare variation of this lesion which included an additional fracture through the scaphoid. Another variant which comprised a fracture dislocation of the elbow with DRUJ dislocation was described by Bock et al^8^ in 1992 and yet a different variant which included a bony injury of the DRUJ was described by Auyeung and Broome^9^ in 2006. However, the extremely rare combination of an Essex-Lopresti lesion and a Galeazzi fracture has only been reported once by Eglseder and Hay^10^ in 1993. A similar injury comprising a posterior dislocation of the radiohumeral joint, a radial shaft fracture, and a DRUJ dislocation was reported by Kedous et al^11^ in 2017. To the best of our knowledge, this is the third case report of this kind.

The management of these injuries remains controversial because there is no clearly defined strategy for the same.^12^ The radial head fractures are usually managed with internal fixation using headless screws and mini-plates or radial head excision or replacement based on the reconstructability of the head. The DRUJ can be stabilized in the acute setting, with a transradioulnar Kirschner wire or a suspensory fixation technique. In the delayed scenario, this is managed with a Sauve-Kapandji or Darrach-type procedure.^13,14^ Some authors also describe methods of reconstructing the interosseous membrane using tendon grafts, although not very popular.^15^ The goal of treatment is to get an accurate reduction of both proximal and DRUJs to reestablish the fulcrum for pronosupination of the forearm. Despite the best efforts at reconstruction, the results are often dismal.

The Galeazzi fracture,^16^ although first described by Sir Astley Cooper in 1822, was named after Riccardo Galeazzi to denote an unstable injury involving the middle third of the radial shaft with a dislocation or instability of the DRUJ. These have been classified into two types by Rettig and Raskin^17^ based on the distance of the fracture line from the articular surface, which is said to have a bearing on the stability of the DRUJ. The instability often goes unnoticed after fixation of the radius, which results in persistent pain and restricted motion. Several Galeazzi equivalents have also been described such as radial shaft fractures associated with physeal separation of the distal ulna in children or a fracture of the distal ulna in adults.^18,19^ A variant of an elbow fracture dislocation combined with a Galeazzi fracture was described by Asadollahi et al in 2013.^20^ The “gold standard” of management of these injuries is an open reduction and stable internal fixation of the radius, followed by assessment of DRUJ stability. If unstable, a transfixation using one or two Kirschner wires is done from the ulna to the radius in a neutral-to-supinated position, taking care to avoid pin placement in the sigmoid notch of the radius. Recently, a method of suspensory fixation of the DRUJ has been described in various studies using mini–plate-button suture loops and adjustable loop endobutton construct (described by the author).^21,22^ In this study, we have used a double endobutton construct with a high–tensile strength suture material such as FiberWire or FiberTape (Arthrex). I would like to clarify here that the term “endobutton” has been used to indicate a fixation device such as a metallic button, over which a suture may be affixed and does not necessarily imply the popular, proprietary device (Smith and Nephew).

The methods for reconstructing the DRUJ are grouped into internal and external approaches. The internal approaches include anatomical reconstruction of the DRUJ ligament, extensor retinaculum with joint capsule reefing, and ulnar wrist suspension tenodesis. The external method uses the elastic properties of a suspensory fixation to externally reconstruct the DRUJ, akin to the syndesmotic tightrope construct used in unstable ankle injuries.^23^ The bony tunnel is made proximal to the sigmoid notch to avoid the synovial joint of the DRUJ. Transradioulnar Kirschner wires require a long period of immobilization, which invariably results in joint stiffness. The benefit of a suspensory fixation over the conventional Kirschner wires is an earlier postoperative recovery and restoration of joint ROM. The low-profile button, which sits flush with the lateral surface of both the bones, does not necessarily require removal, unless symptomatic.

## Conclusion

This study highlights the importance of timely recognition of mutilevel injuries of the wrist and forearm, which may often go unnoticed, thereby compromising the final outcome. A working knowledge of the equivalents or variants of these injuries may be useful in this regard. We also describe a simple and effective technique of stabilization of the DRUJ using a suspensory fixation construct, which may be applied to both acute and chronic injuries.
